# Subclinical Ultrasonographic Abnormalities of the Suspensory Ligament Branches Are Common in Elite Showjumping Warmblood Horses

**DOI:** 10.3389/fvets.2020.00117

**Published:** 2020-03-17

**Authors:** Rachel Mercedes Read, Sarah Boys-Smith, Andrew Perry Bathe

**Affiliations:** Rossdales LLP, Suffolk, United Kingdom

**Keywords:** sports horse, equine, ultrasound, desmopathy, warmblood

## Abstract

**Background:** There is limited information concerning the ultrasonographic appearance of suspensory ligament branches (SLB) in sports horses. Publications exist on clinical injuries that lead to loss of training days and retirement, but not on the appearance of SLBs in high level showjumping Warmbloods.

**Objectives:** To demonstrate the prevalence of subclinical SLB abnormalities in regularly competing high-level showjumpers; to grade each branch 0–3; compare forelimb vs. hindlimb and medial vs. lateral SLBs; subjectively assess periligamentous fibrosis; measure cross sectional area (CSA) and to gather competition follow-up data.

**Study Design:** Prospective cross-sectional study using ultrasonograms.

**Methods:** Sixty elite showjumping Warmbloods without recent history or clinical signs of SLB desmopathy were examined ultrasonographically. Eight static images of each SLB were acquired in transverse and longitudinal sections, anonymised and stored. Images were then assessed and graded by two experienced blinded clinicians based on a published ordinal scale (grade 0–3). A branch score was assigned based on the most severe grade of any image slice in each branch series. SLB cross-sectional area measurement was obtained from each SLB using the transverse image proximal to insertion.

**Results:** The frequency of grade 2 (moderate) ultrasonographic abnormalities was high. Combined data showed a prevalence of 58% (554/960) grade 2 SLBs. Interobserver agreement was good (kappa = 0.65). Periligamentous fibrosis was over represented in hindlimbs (64%). Combined observer data showed there was no statistical difference in branch scores based on limb or laterality. Follow-up over 12 months revealed only two horses were excluded from competition due to SLB injury.

**Main Limitations:** Sample size was small. Images were obtained in static mode, limbs were not clipped of hair, lameness evaluation was under FEI veterinary inspection and not performed by the authors and follow-up evaluation was from FEI competition records and communication only, and was limited to 1 year.

**Conclusions:** Regularly competing elite showjumping Warmbloods have a high prevalence of subclinical SLB ultrasongraphic abnormalities, which may not contribute to causing lameness, poor-performance or be viewed entirely negatively at prepurchase examination. Judicious interpretation of moderate severity SLB abnormalities is advised due the low incidence of clinical injury demonstrated during the 1 year follow-up.

## Introduction

The suspensory ligament is a tendinous band (1) lying deep to the flexor tendons and accessory ligament of the deep digital flexor tendon, palmar/plantar to the third metacarpal/tarsal bones and axial to the second and fourth metacarpal/tarsal bones (2). The suspensory ligament is subdivided into proximal, mid-body, and lateral/medial suspensory ligament branches (SLB) (3). SLBs insert on concave abaxial margins/apices of the proximal sesamoid bones (PSB) and proximal scutum, and a small length lies subsynovially in the metcarpo/tarso phalangeal joint (4). Many publications describe desmopathy of the suspensory origin, with many fewer describing ultrasonography of SLBs (1, 5–9). Branch abnormalities may be linked with lameness, loss of training/competition days, poor performance and loss of sale (3, 5, 10, 11). Lameness and response to diagnostic local analgesia vary in clinical SLB injury, so it's pertinent to describe the ultrasonographic appearance of competing sportshorses. Furthermore, the prevalence of ligament injuries in racehorses have been shown to have a positive correlation with age (12, 13) which may be more considerable in an inherently older population of elite showjumpers.

Due to the high value of elite showjumpers, in-depth pre-purchase examinations include multiple imaging modalities. Radiography is well established in pre-purchase examination, but ultrasonography has only recently become common practice. Limbs are generally unclipped leading to difficult image acquisition, also making experience in interpretation important (14, 15). The appearance of subclinical SLB changes in racehorses have been described (7, 8) but not for sport horses. SLBs have been cited as a frequent site of clinical injury in showjumpers, but it is not expected that data would correlate between racehorse publications and this study involving showjumpers due to differences in age, discipline and training (5, 6, 15, 16).

Both standard and pre-purchase examination of the SLBs involve clinical and dynamic examination, fetlock flexion tests, radiography of the metacarpo(tarso)-phalangeal joint and ultrasonography (3, 11, 17). Ultrasonographic features of injured SLBs include altered shape and size, loss of fibre pattern, hypoechoic lesions, marginal definition loss, hyperechoic foci, entheseous changes and periligamentous fibrosis (1, 3, 5). SLB cross sectional area (CSA) has been established in some breeds (1, 9, 18, 19), however, there is a lack of published normal CSA for Warmbloods.

The aims of this study were to: (1) Investigate the prevalence of subclinical SLB ultrasonographic abnormalities in elite showjumping horses, and grade these abnormalities on 0–3 scale (2) determine the prevalence of ultrasonographic abnormalities in forelimbs vs. hind limbs; and medial vs. lateral SLBs (3) measure CSA proximal to insertion and (4) to collect follow-up data to assess the effect of branch scores on the likelihood of future injury and continued participation in the sport.

## Materials and Methods

### Study Population

Horses were based in the United Kingdom, trained at professional yards and owner permission was granted for their inclusion in this study. Horses were deemed by the owner/trainer to be fit for competition, free of lameness and were competing under Fédération Equestre Internationale (FEI) regulations passing regular veterinary horse inspections. Horses were routinely checked and limbs palpated daily by an experienced trainer/groom. Horse sex, age, and height were recorded from the passports and owner communication. Horses were free from suspensory injury for a minimum of 1 year prior to the date of ultrasonography as declared by the owner/trainer. Competition records from the FEI (www.fei.org) were used to confirm that horses had been competing regularly and at a minimum height of 1.40 m for at least the year prior to the date of ultrasound examination. All horses underwent ultrasonography at a show during competition, and therefore had passed FEI veterinary inspection and were deemed suitable for competition.

### Clinical Examination

Horses were examined clinically by the first author prior to inclusion and ultrasonography. Limbs were palpated in a weight bearing position by running the thumbs and fingers firmly down the dorsal and palmar/plantar borders of the SLBs. Size and pain on palpation were also assessed with the limb held in a flexed position (5, 11). Any horse showing pain on palpation, oedema or palpable enlargement were excluded as these findings have been reported as consistent findings in clinical SLB injury (11).

### Ultrasonography

**Figure 1:**
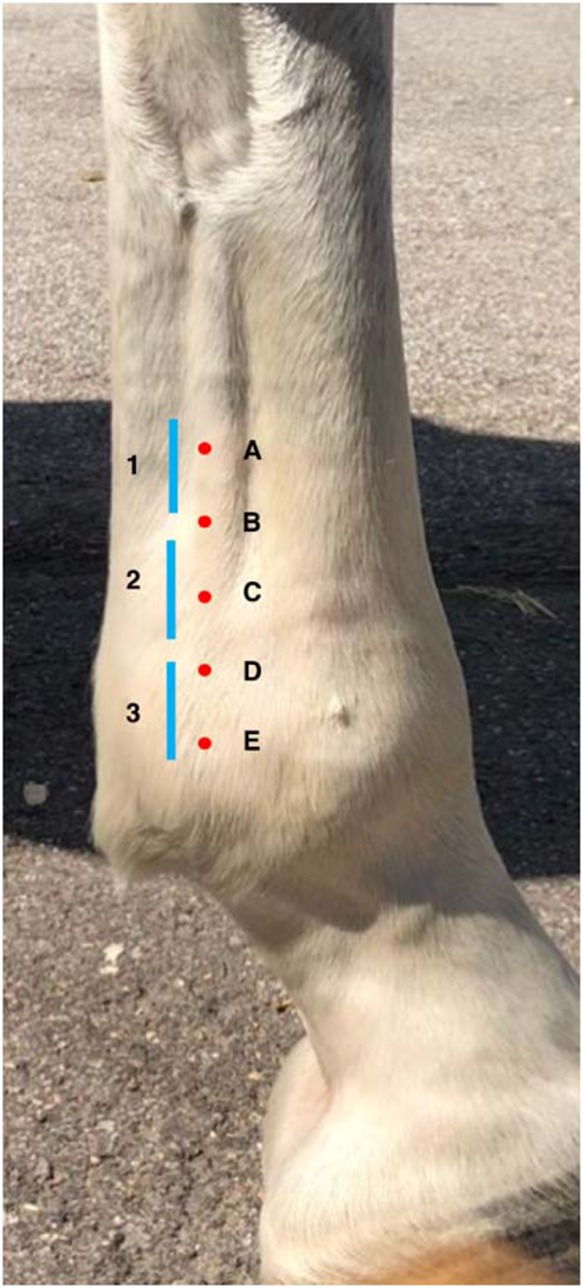
This is a schematic illustration of the location that each of the eight ultrasonogram sections were acquired and stored, where the red dots represent the approximate location of the five transverse sections **(A–E)** proximal to distal and the blue lines represent the approximate location of the and three longitudinal sections, numbered 1–3 proximal to distal. These were acquired based on the approximate distance from the proximal sesamoid bones, however, the entirety of the branch was examined and therefore exact site of the stored images were also adjusted based on ultrasonographic branch shape and appearance.

Ultrasonographic examination of forelimb and hindlimb SLBs was performed by the first author between September 2017 and February 2018. Examinations were performed using a SonoSite M-Turbo® portable ultrasound scanner with a HFL38x 6-13MHz linear array transducer. Horses were not sedated, none of the legs were clipped of hair and imaging parameters were optimised to suit individuals and unclipped limbs. A stand-off pad was not used due to contact issues on the unclipped limbs. The limbs were scrubbed with chlorhexidine, then rinsed with ethanol before an ultrasound coupling gel was applied. Patient identity was anonymised at the time of image acquisition. The order of image acquisition was randomised for each horse, selecting a different limb and branch as a starting point. Horses were stood square and evenly weight bearing, and all suspensory branches were scanned from the level of the bifurcation to insertion on the PSBs from a lateral or medial approach, respectively. A total of eight images were stored per branch: five transverse (starting ~10 cm proximal to PSB then distally at 2 cm intervals to insertion) and three longitudinal (starting ~10 cm proximal to PSB then distally at 4 cm intervals to insertion). Approximate sites of these sections are demonstrated in Figure 1. Due to the variation in height of the horses in this population the exact image position was based on anatomical estimation and ultrasonographic appearance and shape of transverse sections as the SLB transitions from slightly broad elliptical, to round, then triangular and finally flattened ellipse toward the insertion.

### Image Review

Ultrasound images were reviewed subjectively by two independent experienced clinicians, both of whom are both Diplomates in both the European College of Veterinary Surgery and The American College of Veterinary Sports Medicine and Rehabilitation. They were designated as observers “A” and “B.” Observers were blinded to patient identity, limb and laterality. Images were electronically stored in DICOM format then reviewed on the workstation OsiriX. Detailed description on analysis, grading and example ultrasound images in both transverse and longitudinal planes were provided to each observer prior to the start of evaluation. The ultrasonographic appearance for each image acquired was graded on a previously published 0–3 ordinal scale (8), 0—normal (regular echogenicity and fibre pattern); 1—mild hypoechogenicity, subtle irregular fibrillar pattern; 2—extensive regions of mild hypoechogenicity, regions of moderate heterogeneity, small focal disruptions of fibrillar pattern; 3—regions of marked disruption of fibrillar pattern, large anechoic core defect. Fibre/fibrillar pattern in this case refers to the presence or disruption of long linear parallel echoes on the ultrasonograms. A total of 64 image slices were reviewed per horse, eight static slices were graded for each SLB. For further analysis, an overall “branch grade” was then assigned based on the highest grade given to any image slice on the branch. CSA was measured after image storage using the workstation software OsiriX. All branches were measured using the transverse ultrasonogram at the level of the section one slice proximal to PSB insertion. Measurements were recorded in cm^2^ to the third decimal place. Presence of periligamentous fibrosis was also recorded by the observers. The increased periligamentar echogenicity was made subjectively and assessed based on clinician experience and previously published data. It was recorded as increased for a branch if any of the eight sections showed an increase, but was not qualified or quantified beyond noting its presence (5).

### Data Analysis

This is a prospective cross-sectional study using ultrasonograms. Inter-observer agreement was assessed using a weighted kappa statistic. Agreement was considered slight with kappa values between 0 and 0.2, fair 0.2 and 0.4, moderate 0.4 and 0.6, good 0.6 and 0.8, and excellent 0.8 and 1 (7). Wilcox signed rank test was used for comparing medial to lateral, and forelimb to hindlimb. Chi-square statistic and bivariate correlation coefficient tests were used to establish associations between horses' sex, age, height and the number of grade 2 or 3 branches per horse. Descriptive statististics for all CSA measurements were recorded. Spearman's rank correlation coefficient was used to ascertain correlation between reviewer assigned grade and measured CSA. Univariate regression was used to determine relationships between CSA and sex, age and height of the horses. Statistical significance was set with *P* ≤ 0.05.

### Follow-Up

Data were obtained for 12 months following the date of ultrasonography by way of review of competition records and personal communication to track continued participation in the sport at an elite level.

## Results

### Descriptive Statistics

A total of 69 horses were examined. Nine were excluded due to clinical findings indicative of clinical SLB injury. Images of 480 SLBs were acquired in 60 animals. There were 32 males and 28 females, ranging in age from 7 to 17 years (median age of 10 years old). The horses were all Warmbloods, ranging in height from 154 to 184 cm (median 164 cm). All horses were in full competition work at the time of clinical examination and ultrasonography.

### Inter-Observer Agreement

There was agreement in SLB grading in 396/480 (82.5%) of branches and inter-observer agreement was considered good (kappa 0.65; 95% CI 75.8–85.8). Inter-observer agreement for grade 3 lesions was excellent (kappa 0.83).

### Grades

**Figure 2:**
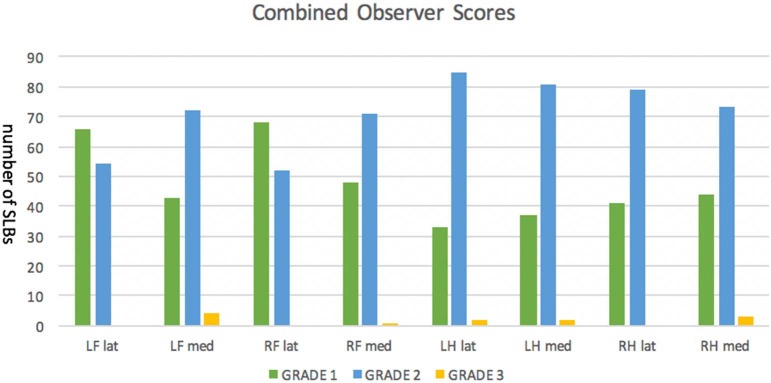
Suspensory ligament branches (SLB) in a population of 60 elite level showjumpers including forelimb (FL) and hindlimb (HL), and medial (med) and lateral (lat) branches were evaluated ultrasonographically, and eight images were stored for each branch. This figure shows the combined SLB scores of two blinded observers using a 0–3 ordinal scale. There were no SLBs with an overall branch score of 0.

Combined data showing prevalence of each branch grade is represented in Figure 2, which demonstrates the frequency of grades for each individual observer, as well as where there was agreement. These grades are the overall branch scores, which have been assigned based on the worst grade given to an ultrasonogram slice in that branch. All but one horse had at least one branch that was grade 2. Grades given by observers A and B have been summarised and combined in Table 1: grade 0 0/960 (0%), grade 1 394/960 (41%) and, grade 2 554/960 (58%), and grade 3 12/960 (1%). Examples of each grade from horses in this study are shown in Table 2. The median number of grade 2 branches per horse for observer A was 4/8, and observer B was 5/8. The distribution in grades between forelimb vs. hindlimb SLBs was significantly different for both observers (observer A *P* = 0.001 and observer B *P* = 0.002). More grade 2 and 3 abnormalities were recorded in hindlimb SLBs. Combined data showed 255/480 (53%) of forelimbs and 321/480 (67%) of hindlimbs were graded 2 or 3. Comparison was also made for laterality in SLBs graded 2 or 3: 117/240 (48.7%) lateral and 131/240 (54.5%) medial for observer A, vs. 148/240 (61.1%) lateral, and 170/240 (70.8%) medial for observer B. The prevalence of grade 2 or 3 SLBs was higher in medial branches for both observers, but was only statistically significant for observer B (*P* = 0.026), not for observer A (*P* > 0.05).

**Table 1:** The frequency of suspensory ligament branch (SLB) grades given by independent evaluation of two blinded observers (observer A and B) are shown.

**Observer scores**						
		**Observer B**				
		Grade 0	Grade 1	Grade 2	Grade 3	Total (observer A)
**Observer A**	Grade 0	0	0	0	0	**0**
	Grade 1	0	156	76	0	**232**
	Grade 2	0	6	235	2	**243**
	Grade 3	0	0	0	5	**5**
	Total (observer B)	**0**	**162**	**311**	**7**	960

*These branch grades were assigned to each SLB based on eight static image slices of each of 480 SLBs in a population of 60 elite level showjumping warmbloods. Grades are combined to show both the total number of SLBs for each grade by each observer and also frequency of agreement and disagreement between the observers*.

**Table 2:** Example ultrasonograms in transverse and longitudinal for grade 0–3 branches taken of SLBs from horses included in this study.

**Example ultrasonograms graded using a 0-3 ordinal scale**
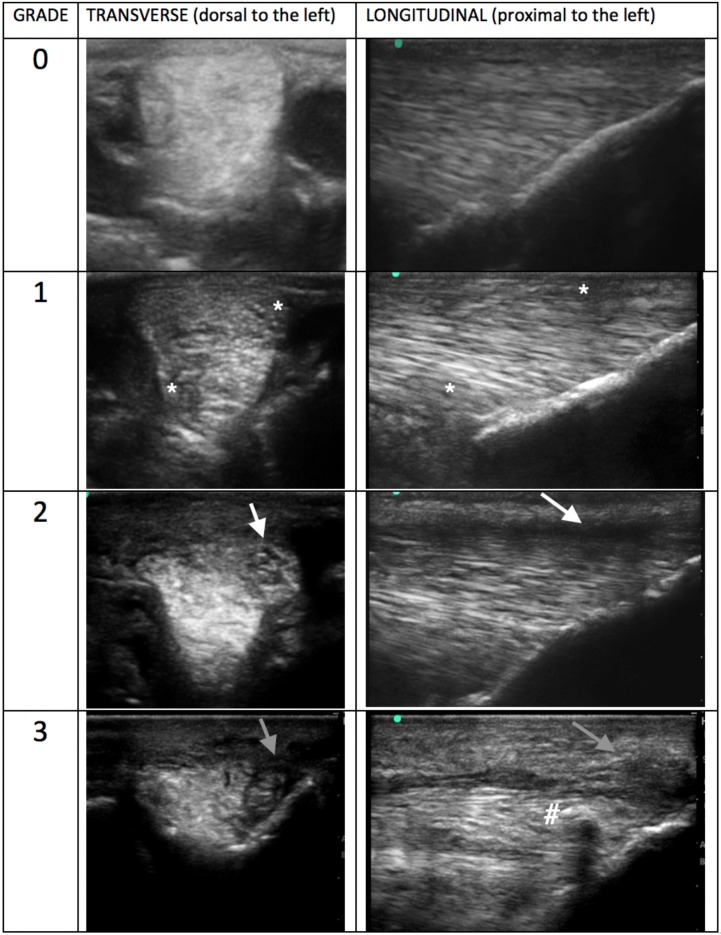

*These images are taken at or just proximal to insertion on the proximal sesamoid bones. Grades were assigned using a previously published ordinal 0–3 scale (8) where here, 0 is normal showing regular echogenicity and fibre pattern; 1 shows mild hypoechogenicity and subtle irregular fibrillar pattern (white asterisk); 2 has more extensive regions of mild hypoechogenicity and moderate heterogeneity (white arrow); 3—regions of marked disruption of fibrillar pattern (grey arrow) and mineralisation (white hash)*.

### Cross-Sectional Area

The mean measurements in this population for SLB cross-sectional area in cm^2^ with standard of deviations are displayed in Table 3. The forelimb medial branches showed a slightly larger CSA then forelimb lateral branches, as did both medial and lateral hindlimb SLBs compared to forelimb lateral branches. The data shows no significant difference in laterality comparison between medial and lateral in the hindlimb SLBs. There was also no significant association between assigned SLB grades, sex, age, or height and the CSA proximal to insertion.

**Table 3:** Mean cross-sectional areas (CSAs) of suspensory ligament branches (SLBS) in a population of elite level showjumping warmbloods with no recent history or current clinical signs of clinical SLB injury.

**Cross-sectional area measurements of suspensory ligament branches**	
	**Mean** **±** **s.d CSA**
**SLB**	**CSA SLBs in cm**^**2**^ **proximal to insertion**
Left forelimb lateral	1.412 ± 0.173
Left forelimb medial	1.505 ± 0.159
Right forelimb lateral	1.417 ± 0.154
Right forelimb medial	1.528 ± 0.149
Left hindlimb lateral	1.512 ± 0.197
Left hindlimb medial	1.570 ± 0.199
Right hindlimb lateral	1.505 ± 0.198
Right hindlimb medial	1.561 ± 0.188

*CSA measurements were based on stored transverse images taken ~2 cm proximal to insertion on the proximal sesamoid bones (level D on Figure 1)*.

### Periligamentous Fibrosis

**Figure 3:**
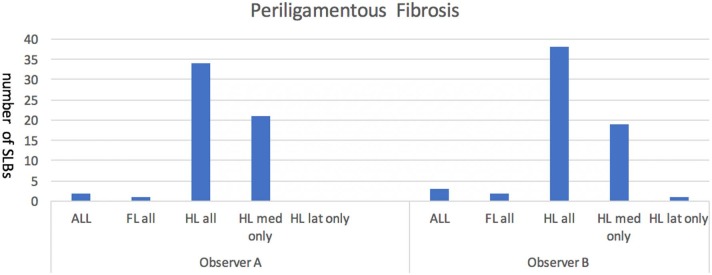
Represents the number of forelimb (FL) and hindlimb (HL), medial (med) and lateral (lat) suspensory ligament branches (SLBs) where the presence of periligamentous fibrosis was noted by the observers to be increased compared to normal. Periligamentous fibrosis was noted to be most frequently associated with the hindlimb SLBs in this population of elite level showjumpers. There was no significant difference noted between forelimb medial or lateral branches, but a significant difference was noted with hind limb medial branches showing an increase in fibrosis when compared to both hindlimb lateral or any forelimb branches.

Combined data demonstrated presence of periligamentous fibrosis in 7% of forelimb, and 64% of hindlimb SLBs. More specifically, the hindlimb medial branches were most frequently associated with periligamentous fibrosis, these data are demonstrated in Figure 3. Ultrasonograms using horses in this study exemplifying absence vs. presence of fibrosis and are shown in Table 4.

**Table 4:** Comparison ultrasonograms taken from a population of elite level showjumping Warmbloods having no recent history or clinical signs of suspensory ligament branch (SLB) injury.

**Normal vs. increased periligamentous fibrosis**
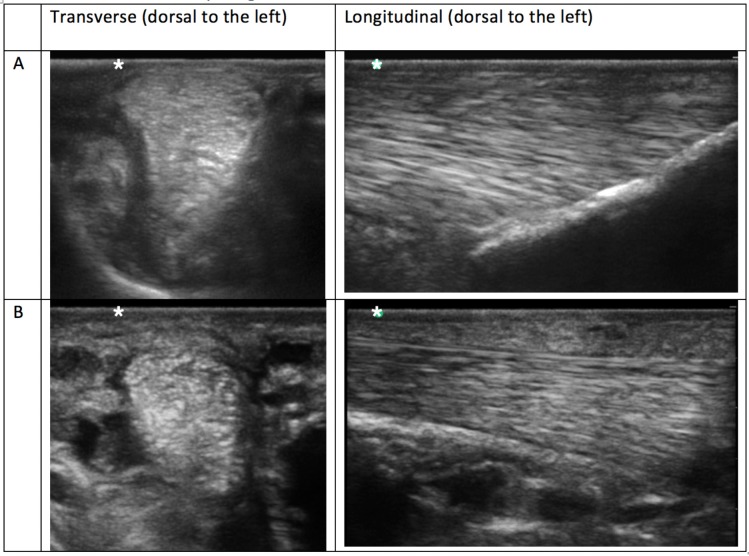

These example ultrasonograms are used to show what observers deemed as normal, (A) vs. subjectively increased (B) amounts of periligamentous fibrosis (PF) lying deep to the skin (white asterisk*) and adjacent to the SLB.

### Age, Sex, Height

There was no significant statistical difference between scores given by either observer in relation to the age, sex or height of the horses.

### Follow-Up

Data was obtained for all but two horses (58/60) following the sale of those animals. Eighty-four percent of horses (49/58) continued to compete at an elite level on a minimum of 10 occasions. Of the nine that did not, seven were for reasons unrelated to SLB injury. Two were due to lameness, one due to sudden cardiac death, and four were under new riders competing at a lower level. There were only two horses reported to be out of competition due to suspensory injury. One which involved hindlimb proximal suspensory desmitits and a hind limb medial SLB injury: that branch had been graded in this study as only grade 1 by both observers prior to injury. The other was due largely to isolated front foot related lameness, but a forelimb lateral branch injury was also identified by the attending veterinarian: that branch had been graded as a 2 by both observers.

## Discussion

We are not aware of any previous publications which outline the prevalence of subclinical ultrasonographic abnormalities of SLBs in elite performing showjumpers. The high frequency at which the moderate severity ultrasonographic changes were recognised in this population of regularly competing showjumpers may aid in clinical image interpretation. Most horses (59/60) had at least one SLB with a grade 2 ultrasound abnormality. This is significant as Grade 2 changes were of a severity that all authors agreed could have previously been considered clinically significant, therefore, the high frequency which we have shown them to be present in these showjumpers considered to be fit for competition was surprising. Published data using the same scale in grading clinical injury of SLBs did report that 11.8% of clinical SLB injuries showed grade 2 changes, with the other 88.2% grade 3 (5). Additional published work has also shown that ultrasonographic SLB abnormalities are common, albeit milder, in non-lame control horses when compared with lame horses where lameness was isolated to the region of the SLBs. (20) It was agreed by all authors that Grade 0 and 1 ultrasonographic abnormalities would not be considered clinically significant. It was further agreed that grade 3 abnormalities would be considered in practice to be clinically significant and contribute to a high-risk purchase.

Prepurchase examination now frequently includes SLB ultrasonography and evidence of branch alteration can lead to a horse being considered unsuitable for purchase. Grade 2 abnormalities were previously considered a risk to purchase and future injury. However, in short term follow-up in a similar study on National Hunt racehorses no horses with grade 2 SLBs developed SLB injury in the following season and were therefore confirmed as subclinical changes (7). The same can be considered true in this study population, as Grade 2 changes were more prevalent, especially in hind limbs. These seemed to be tolerated well, as only one horse with a Grade 2 change was reported to develop clinical SLB injury.

Grade 2 and 3 abnormalities were significantly more common in hindlimbs vs. forelimbs, which is not consistent with previously published data on frequency of clinical injury (5) where there was no significant difference shown between forelimbs and hindlimbs. Also, structural tissue type, quantity of collagen and muscle tissue is naturally symmetrical between thoracic and pelvic limbs (21). Similar studies published on subclinical ultrasonography for both Flat and National Hunt racehorses (7, 8) did not include ultrasonography of the hindlimbs, and as such, no data exists on subclinical abnormalities in forelimbs compared to hindlimbs. Hindlimb proximal suspensory desmopathy has also been shown to be one of the most frequently recorded injuries in elite level showjumpers (6), and forelimb soft tissue injuries are found more commonly in the superficial and deep digital flexor tendons (6). It follows that the hindlimb suspensory ligaments may be under more strain and therefore the SLBs show more advanced subclinical changes. Combined observer data showed no significant difference in grades assigned to medial vs. lateral branches. Clinical injury in sports horses has also not been shown to be more frequent in medial vs. lateral branches (5). Conversely, published data on Flat (7) and National Hunt (8) racehorses had both shown that there is a significantly higher incidence in changes in the medial branches, so this is likely discipline dependent, potentially due to the fact that medial limb loading pathology is seen more frequently in sports involving galloping.

There was a high frequency of periligamentous fibrosis associated with the hindlimbs, specifically hindlimb medial branches. Previous work on clinical SLB injuries in sports horses noted a similar trend, recording periligamentous fibrosis in 78.7% of hindlimbs (5). Consideration should be made when palpating and ultrasonographically assessing hindlimbs because this increase could be associated with chronic SLB injury preceded by cumulative degenerative changes (1, 22) but is also noted to be present frequently in this population which have been considered subclinical.

We have shown no statistical difference in the number of grade 2 or 3 branches or CSA in the older vs. younger horses in this population. Thoroughbred racehorses have been shown to have a rate of suspensory ligament injury 5-fold more frequent in 5-year-olds, than 2-year-olds (13) and a higher incidence of mild to catastrophic SLB injury is reported for racehorses older than 5-years-old (23). On necropsy, moderate suspensory apparatus injury was a common finding in the racehorses older than 7 years (24). In Standardbred racehorses functional integrity has been shown to decrease with age, repetitive loading, and chronic inflammation (21), this may also be true in other breeds and disciplines. It was also previously speculated when comparing subclinical ultrasonography of older National Hunt vs. younger Flat racing thoroughbreds that the reason for the huge difference between the number of grade 2 changes (30.6 and 6.7%, respectively) was due to age and training years (7). In that group of National Hunt racehorses, the mean age was 5 years old, whereas in this group of showjumpers the youngest horse was seven, so it can be assumed that older age of this entire population, along with breed and discipline account for the different frequency of changes observed.

Unspecific to SLBs, there is structural and component variation between tendon and ligament tissue in males vs. females. Connective tissue percentage is higher in males, and muscle tissue is significantly higher in females, both of which are likely the result of hormonal influences (21). Despite these structural variances, there was no difference between branch grades and sex in this population.

It has been previously reported that there is a lack of correlation in branch cross-sectional area (CSA) and branch grade in National Hunt horses (7), and the same was true in this population. Breed standards have been published for Flat Thoroughbred, Arabian, and Spanish horses (9, 18, 19), but there is an absence of published data on normality for Warmbloods. The mean CSA for horses in this study was higher than that published in these other breeds. The greater CSA measurements may be adaptive and due in part to the comparatively larger size and stature of some Warmbloods, but the horses in this study cannot be considered breed standard for Warmblood horses due to the intensity of training they undergo. The increase in CSA measurements cannot be directly compared to other publications using different populations, and more work is required in order to determine what influence breed, age and type and intensity of training has. CSA has been shown to be greater in the medial compared to lateral branches (1, 9), which was corroborated for forelimbs only in this study, but this is not considered clinically relevant as this did not correlate independently with more severe branch scores.

Limitations encountered in this study were noted during limb preparation and at image acquisition. Limbs were not clipped of hair and horses were not sedated, resulting in decreased image quality due to horse movement and the coarser distal limb hair that many Warmbloods have. Limb preparation in this case can be compared to real world circumstances, as owners usually have a strong desire not to clip horses who otherwise may be fit for competition or sale. Assessment of each SLB was made on eight static images: diagnosis based on real-time ultrasound is more apt to accurate representation, however, image acquisition was carried out by an experienced orthopaedic clinician and it is therefore more likely that the images acquired were of good quality and representative of the SLBs (25). Authors also agreed that static image review contributed to problems with interpretation due to artefact and angle of incidence resulting from the varied fibre angle alignment in the distal branch. Periligamentous fibrosis was estimated by objective assessment only and thickness was not measured. Furthermore, because a standoff pad was not used differentiation between skin and fibrosis may have been more difficult. All horses underwent thorough limb palpation in order to exclude horses showing signs of clinical injury, however, lameness evaluation was not performed by the first author as it is considered an inconsistent feature of, and non-specific for SLB desmopathy. However, horses that were included had all passed FEI veterinary inspection during the same week and prior to ultrasonography, and therefore were considered fit for competition. Finally, follow-up data was limited to 1 year and only gathered in the form of competition results and personal communication. The horses were not necessarily reexamined physically or ultrasonographically by the first author.

Thirteen percent (9/69) horses examined for this study showed clinical evidence and/or history of SLB injury within the previous year, and were therefore excluded. This is a similar rate to detection in Thoroughbreds where an estimated 14% of racehorses in training have a palpable forelimb suspensory apparatus injury (24) and another study showed 22.5% of race starters in Kentucky had palpable abnormalities of the SLBs (26). There is such a disparity in the high number of grade 2 subclinical SLB scores in this population of elite showjumpers, compared to the very low numbers in Flat racehorses (8) especially in contrast to the very similar numbers of palpable clinical lesions between the same two populations (23, 26). We speculate this is due to the fact that micro damage of suspensory ligaments accumulates over athletic lifetime (24) and that there is therefore likely to be a persistence of ultrasonographic abnormalities beyond clinical normality (10). The addition of power Doppler may be useful in further assessment of the chronicity or severity of abnormalities detected in B-mode (20). Due to the long careers that showjumpers have and the lack of a distinct off season, many do have cumulative damage that persists ultrasonographically while successfully remaining in work. Monitoring these limbs clinically is made difficult as to differentiate between clinical and subclinical lesions by palpation and lameness alone is unreliable, especially if partial tears are present (5, 10).

## Conclusions

This study revealed that elite level showjumpers often show Grade 2 ultrasonographic SLB abnormalities. Hindlimb periligamentous fibrosis was also subjectively observed at a frequency similar to that previously published to be associated with clinical injury, although the exact thickness of which was not measured. The frequency of grade 2 or 3 abnormalities was much higher in this population than that published for young racehorses, which may be due to the older age of this population and cumulative damage over a year-round and comparatively lengthy competitive life. Follow-up of the horses in this study limited to 1 year showed a low risk of SLB injury associated with the frequently identified moderate severity subclinical changes. Therefore, when Grade 2 changes are detected in competing elite level showjumpers which lack palpable abnormality, they may not be clinically relevant and carry a low risk of future injury (3%), when compared to the rate of detection of SLB injury in the same competing population (13%). Further work including advanced imaging of SLBs using off incidence and power Doppler ultrasonography, elastography and MRI maybe be useful in further characterizing SLB abnormalities.

## Data Availability Statement

The data that support the findings of this study are openly available in Figshare at http://figshare.com/articles/branch_scores/7987175. Further raw data supporting the conclusions of this article will be made available by the authors, without undue reservation to any qualified researcher.

## Manufacturers Addresses

Minitab Inc., State College, Pennsylvania, USA. Osirix Imaging Software, https://www.osirix-viewer.com/ SonoSite Inc., Bothwell, Washington, USA https://data.fei.org/Horse/Search.aspx.

## Ethics Statement

Ethical review and approval was not required for the animal study because owner consent was given to perform ultrasonography in order that they gain further information into the health of their animal, and gave permission alongside this to use the information in publication. No invasive techniques were used. Written informed consent for participation was not obtained from the owners because oral consent was provided by an owner or the managing connections of the animal as most of the horses were professionally ridden and trained, and under the care of these professionals.

## Author Contributions

RR was involved in study design, acquired the images, and was responsible for data analysis. AB and SB-S graded the images. All authors contributed to preparation of the manuscript.

### Conflict of Interest

The authors declare that the research was conducted in the absence of any commercial or financial relationships that could be construed as a potential conflict of interest.
